# Dual Defect-Engineered BiVO_4_ Nanosheets for Efficient Peroxymonosulfate Activation

**DOI:** 10.3390/nano15050373

**Published:** 2025-02-28

**Authors:** Jiabao Wu, Meiyu Xu, Zhenzi Li, Mingxia Li, Wei Zhou

**Affiliations:** 1 School of Chemistry and Materials Science, Key Laboratory of Functional Inorganic Material Chemistry, Ministry of Education of the People’s Republic of China, Heilongjiang University, Harbin 150080, China; wjb2201867430@163.com; 2 Shandong Provincial Key Laboratory of Molecular Engineering, School of Chemistry and Chemical Engineering, Qilu University of Technology (Shandong Academy of Sciences), Jinan 250353, China; 18053165243@163.com (M.X.); zzli@qlu.edu.cn (Z.L.)

**Keywords:** photocatalysis, defect engineering, doping engineering, peroxymonosulfate, ciprofloxacin degradation

## Abstract

Defects and heteroatom doping are two refined microstructural factors that significantly affect the performance of photocatalytic materials. Coupling defect and doping engineering is a powerful approach for designing efficient photocatalysts. In this research, we successfully construct dual defect-engineered BiVO_4_ nanosheets (BVO-N-OV) by introducing N doping and oxygen vacancies through ammonium oxalate-assisted thermal treatment of BiVO_4_ nanosheets. Due to the combined enhancement of band structure and surface properties from N doping and oxygen vacancies, the obtained BVO-N-OV nanosheets demonstrate improved visible light absorption, effective charge transfer efficiency, and increased active sites. As a result, the constructed BVO-N-OV/PMS system demonstrates significantly enhanced ciprofloxacin (CIP) removal performance under visible light illumination. The highest rate constant for CIP degradation over BVO-N-OV/PMS system is 7.9, 1.9, and 6.6 times greater than pristine BiVO_4_ (BVO), oxygen vacancy-enriched BiVO_4_ (BVO-OV), and N-doped BiVO_4_ (BVO-N), respectively. Even in a broad pH range (3.0–11.0) with various anions, the BVO-N-OV/PMS/Vis system still demonstrates stable and excellent CIP removal performance. This study seeks to provide valuable insights into the interaction between defect and doping engineering in photocatalytic activation of PMS, thereby proposing new strategies for designing effective photocatalyst/PMS systems for wastewater treatment.

## 1. Introduction

Antibiotics and other persistent, refractory organic pollutants are present in various water environments, posing a serious threat to ecosystems and human health [1,2]. Therefore, it is urgent to explore a green and efficient strategy for the removal of pollutants from water. The peroxymonosulfate-based advanced oxidation process (PMS-AOPs) can generate various highly oxidative reactive oxygen species (ROS) to break down organic contaminants, thus making it a promising technology for organic wastewater treatment [3,4,5]. However, the O-O bond in PMS has a bond length of 1.453 Å with a bond energy of 377 kJ/mol, suggesting the activation of PMS is challenging [4,6]. Among the various PMS activation strategies, photocatalysis has attracted significant interest because of its benefits such as energy conservation, sustainability, and environmentally friendly, making it a hot topic in current research [7]. However, most of the developed photocatalysts face issues like low solar light absorption efficiency, severe charge recombination, and insufficient reactive active sites, leading to inefficient PMS activation processes and a significant gap in many practical requirements [8]. Therefore, it is of great significance to conduct in-depth research on the influence of structure on the performance of photocatalytic materials and to develop efficient and stable photocatalysts for PMS activation.

The catalytic efficiency of photocatalytic materials is closely related to numerous structural factors. Defects and heterogeneous atom doping are two key microscopic structural factors that affect the performance of photocatalytic materials. Through precise calculations and simulations, density functional theory (DFT) modeling can reasonably design operations such as nitrogen doping and defect formation, providing important theoretical support for experiments, and has been validated in numerous studies in recent years [9,10]. On one hand, the presence of defects is a common phenomenon in material synthesis of photocatalytic nanomaterials. Defects can effectively regulate the performance of photocatalysts. For example, oxygen vacancy defects, which are commonly found in metal oxides, can optimize conductivity and band structure, thereby significantly enhancing photon absorption, exciton generation, and carrier separation processes. More importantly, oxygen vacancy defects act as highly active reaction sites, promoting the adsorption and activation of substrate molecules [11,12,13]. Especially for PMS activation reactions, the highly symmetric ion distribution on both sides of the oxygen vacancy is beneficial for the lateral adsorption of the two oxygen atoms in the O-O bond of the PMS molecule onto the catalyst. In addition, the abundant electrons confined by the oxygen vacancy can accelerate the interfacial electron transfer between the PMS molecules and the catalyst, thus promoting PMS activation [14,15,16]. Therefore, further research on the impact of defects on photocatalytic PMS activation will be beneficial for constructing efficient and stable photocatalytic PMS activation systems. Moreover, impurity energy levels will be generated within the semiconductor’s band gap due to the introduction of heteroatoms, thereby narrowing the band gap and broadening light absorption. Also, heteroatom doping leads to an uneven distribution of charges, thereby creating an internal electric field that facilitates the migration and separation of photo-generated charge carriers [17,18]. For PMS activation reactions, heteroatom doping will create numerous asymmetric sites, thus reducing the energy barrier for PMS adsorption and activation on the photocatalyst surface and accelerating the breaking of the O-O bond in PMS and the production of reactive oxygen species (ROS) [19,20]. Both defect engineering and doping engineering can regulate photocatalytic PMS activation reactions. Actually, during the semiconductor doping process, the introduction of dopants is often accompanied by the generation of defects [21,22,23]. In other words, defects and heteroatoms may interact with each other during the photocatalytic PMS activation process. Studies have shown that coupling heterogeneous atoms and vacancy defects is an effective strategy to improve photocatalytic performance. For example, Zhang et al. [24] developed a Nitrogen-doped BiOCl atomic layers photocatalyst, homogeneously doped N and co-engineered OV reconstructed the electronic structure of BiOCl, thus kinetically facilitating CO_2_-to-CO reduction. However, whether coupling defects and heteroatoms can promote the PMS activation for pollutants degradation remains to be studied. Systematically studying the combined enhancement of defect and doping engineering on photocatalytic PMS activation performance will promote the understanding of the essence of photocatalysis and the development of efficient photocatalytic materials.

Two-dimensional (2D) layered nanomaterials have been extensively investigated in photocatalysis due to the large specific surface area and the short bulk carrier migration distance [25,26,27,28,29,30,31,32,33,34]. More importantly, compared to the corresponding bulk materials, the surface atoms of 2D ultrathin nanosheets have lower escape energy, making them more easily replaced by heteroatoms or escape from the lattice [35]. Therefore, 2D ultrathin nanosheets can serve as an ideal structural model for defect engineering and doping engineering.

In this study, layered BiVO_4_ is employed as a model material to validate this concept. By performing ammonium oxalate-assisted post-heat treatment on BiVO_4_ nanosheets, nitrogen doping and oxygen vacancy dual defects were created. The structure, photoelectrochemical properties, and photocatalytic performance of the resulting catalysts were characterized and evaluated in detail. Experimental results show that dual defects synergistically optimize the band structure, enhance light absorption, and promote the migration and separation of photo-generated carriers. In addition, the introduction of oxygen vacancies and nitrogen doping optimized the surface properties, thereby providing abundant sites for the adsorption and activation of PMS molecules. Consequently, compared to the pristine BiVO_4_ (BVO), oxygen-deficient BiVO_4_ (BVO-OV), and N-doped BiVO_4_ (BVO-N), the dual defect-engineered BiVO_4_ nanosheets (BVO-N-OV) exhibited significantly enhanced PMS activation performance under visible light irradiation. This work is anticipated to offer valuable insights into the coupling mechanism of defect engineering and doping engineering in photocatalytic PMS activation, and to propose new strategies to enhance the photocatalytic efficiency of materials through microstructure regulation.

## 2. Experimental Section

### 2.1. Materials

All materials used in this work are commercially available and used without any treatment. The purchased chemicals and the corresponding providers are listed as follows: Bismuth nitrate pentahydrate (Bi(NO_3_)_3_⋅5H_2_O 99%, Aladdin, Shanghai, China), Ammonium metavanadate (NH_4_VO_3_ 99%, Sinopharm, Beijing, China), Sodium dodecylbenzenesulfonate (C_18_H_29_NaO_3_S 95%, Aladdin, Shanghai, China), Oxalic acid (C_2_H_2_O_4_ 99%, Aladdin, Shanghai, China), Ammonium oxalate monohydrate (C_2_H_8_N_2_O_4_⋅H_2_O AR, Sinopharm, Beijing, China), Sodium hydroxide (NaOH 99%, Sinopharm, Beijing, China), Nitric Acid (HNO_3_ AR, Kermel, Tianjin, China), Potassium hydrogen monnopersulfate (KHSO_5_⋅0.5KHSO_4_⋅0.5K_2_SO_4_ 99%, Aladdin, Shanghai, China), Ciprofloxacin (C_17_H_18_FN_3_O_3_ 98%, Macklin, Shanghai, China), Ethanol (C_2_H_5_OH 99%, Sinopharm, Beijing, China).

### 2.2. Synthesis

*Preparation of BiVO_4_*: The flake-like BiVO_4_ was synthesized using a hydrothermal method. 0.57 mmol of sodium dodecylbenzenesulfonate (SDBS) and 2.0 mmol of Bi(NO_3_)_3_·5H_2_O were dissolved in 10 mL of 4M HNO_3_ aqueous solution and stirred for 1 h. 2.0 mmol of NH_4_VO_3_ were dispersed in 10 mL of 2 M NaOH aqueous solution, Then, the above solution was added to the Bi(NO_3_)_3_·5H_2_O solution under stirring for 0.5 h. Next, 2 M NaOH were dropwisely added into the above solution to adjust the pH of the mixture to 6–7 under stirring for 0.5 h. Then, the mixed solution was transferred to a 100 mL stainless steel autoclave (PTFE-lined) and hydrothermal treated at 200 °C for 2 h. After cooling to room temperature, the products were washed with deionized water and ethanol and dried at 80 °C for 8 h.

*Preparation of BiVO_4_-OV*: Completely mixed 100 mg of the original BiVO_4_ nanosheets with 150 mg of oxalic acid. The above mixture was placed in an Ar atmosphere and heated it to 280 °C at a rate of 2 °C/min, maintained for 2 h. Subsequently, the samples were washed with anhydrous ethanol to eliminate any remaining impurities. Lastly, the samples were vacuum-dried at 60 °C for 8 h until completely dry. 

*Preparation of BiVO_4_-N*: 100 mg of the original BiVO_4_ nanosheets were heated in an NH_3_ atmosphere at a rate of 2 °C/min to 280 °C, and maintained for 2 h.

*Preparation of BiVO_4_-N-OV*: Completely mixed 100 mg of the original BiVO_4_ nanosheets with 170 mg of ammonium oxalate monohydrate. The mixture was placed in a muffle furnace and heated to 280 °C at a rate of 2 °C/min, then maintained at 280 °C for 1.5 h, 2 h, and 2.5 h, respectively. Subsequently, the samples were washed with anhydrous ethanol to eliminate any remaining impurities. Lastly, the samples were vacuum-dried at 60 °C for 8 h until completely dry.

## 3. Results and Discussion

**Scheme 1:**
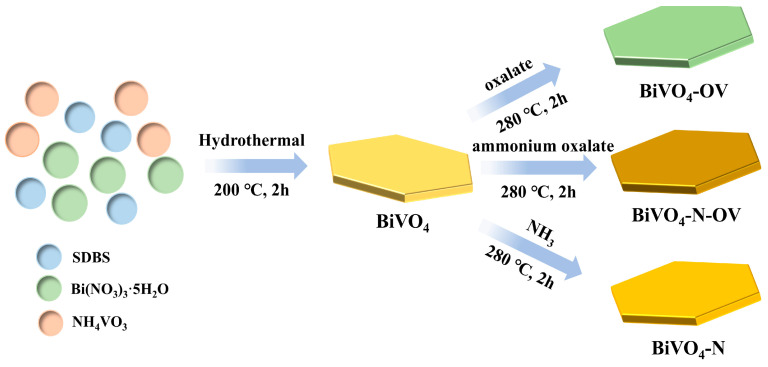
Schematic diagram of the synthesis of BiVO_4_, BiVO_4_-OV, BiVO_4_-N and BiVO_4_-N-OV.

**Figure 1:**
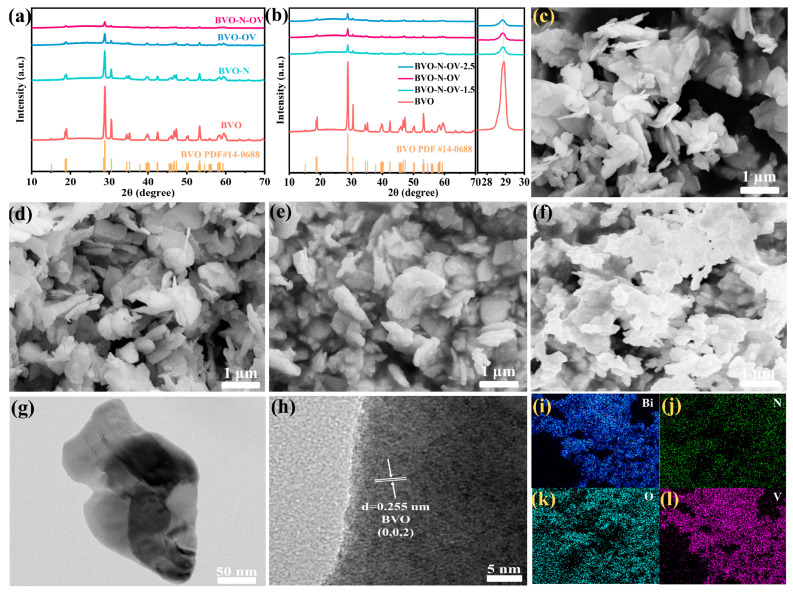
XRD patterns of the (**a**) BVO, BVO-N, BVO-OV and BVO-N-OV; (**b**) BVO and BVO-N-OV-1.5, BVO-N-OV, BVO-N-OV-2.5; SEM images of (**c**) BVO; (**d**) BVO-N; (**e**) BVO-OV; (**f**) BVO-N-OV; (**g**) TEM, (**h**) HRTEM and (**i**–**l**) EDX elemental mapping profiles of BVO-N-OV.

Scheme 1 depicts the preparation method of the dual defect-engineered BiVO_4_ nanosheet photocatalyst. Initially, BiVO_4_ nanosheets were prepared through a hydrothermal process. Subsequently, the dual defect-engineered BiVO_4_ nanosheets were obtained by annealing the BiVO_4_ nanosheets with ammonium oxalate assistance. For comparison, N-doped BiVO_4_ nanosheets were prepared by annealing the BiVO_4_ nanosheets in an NH_3_ atmosphere. Meanwhile, oxygen vacancy-rich BiVO_4_ nanosheets were synthesized by oxalate-assisted annealing of BiVO_4_ nanosheets in an inert atmosphere. The X-ray diffraction (XRD) patterns of the synthesized BVO-N-OV, BVO, BVO-N, and BVO-OV samples well matched with monoclinic BiVO_4_ (JCPDS: 14-0688) (Figure 1a) [36]. In the XRD patterns of the obtained materials, no additional diffraction peaks other than those of BiVO_4_ were observed, indicating the high purity of the synthesized samples. In addition, as the amount of N doping increases, the XRD diffraction peak corresponding to the (121) plane at 28.9° gradually shifted (Figure 1b), indicating that N atoms were successfully integrated into the BiVO_4_ lattice or substituted O atoms [37]. Scanning electron microscope (SEM) images revealed that the pristine BiVO_4_ displayed a thin, smooth-surfaced nanosheet morphology (Figure 1c). After introducing N doping and oxygen vacancy defects, the morphology and dimension of the BiVO_4_ nanosheets remained largely unchanged (Figure 1d-f). In addition, transmission electron microscope (TEM) and high-resolution TEM (HR-TEM) images (Figure 1g,h) further validated the nanosheet morphology of the dual defect-engineered BiVO_4_. The lattice fringe spacing of 0.255 nm aligns with the (002) plane of monoclinic BiVO_4_. The lattice spacing value of the (002) crystal plane of the dual defect-engineered BiVO_4_ nanosheets was similar to that of the pristine BiVO_4_, likely due to the similar radii of N atoms (0.75 Å) and O atoms (0.73 Å). In addition, as shown in Figure 1i–l and Figure S1, EDX element mapping confirmed N incorporation and indicated that N, O, Bi, and V elements were uniformly spread across both BVO-N and BVO-N-OV.

**Figure 2:**
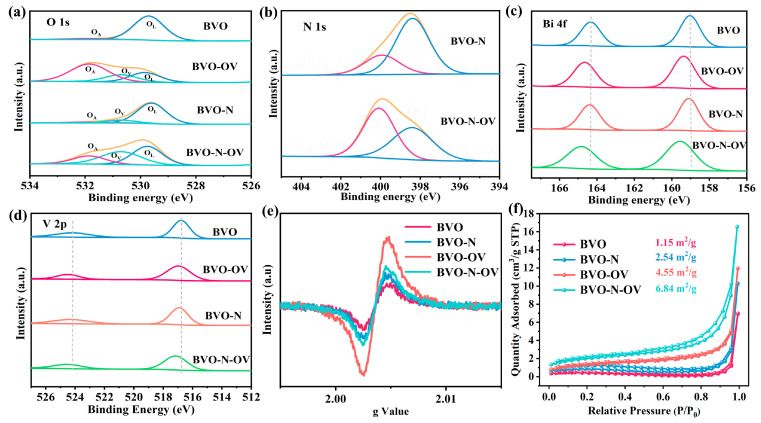
XPS spectra of BVO, BVO-OV, BVO-N, BVO-N-OV (**a**) O 1s; (**b**) N 1s; (**c**) Bi 4f; (**d**) V 2p; (**e**) EPR spectra; (**f**) N_2_ adsorption-desorption isotherms of BVO, BVO-N, BVO-OV and BVO-N-OV.

X-ray photoelectron spectroscopy (XPS) was further employed to examine the impact of N doping and oxygen vacancy defects on the chemical composition and surface chemical states of the BiVO_4_ nanosheets. The O 1s XPS spectra of the samples (Figure 2a) displayed two peaks around 529.7 and 531.9 eV for the BVO sample, attributed to lattice oxygen atoms (O_L_) and surface-adsorbed oxygen species (O_A_), respectively. In contrast, the O 1s XPS spectra of the BVO-OV, BVO-N, and BVO-N-OV samples showed a new peak at approximately 530.7 eV, which could be attributed to defect-state oxygen. The result proved the presence of oxygen defects in the samples [38,39]. Moreover, the O 1s XPS peak at 530.7 eV for the BVO-N-OV sample exhibited a larger peak area compared to the BVO-OV and BVO-N samples, indicating that the BVO-N-OV sample had a higher concentration of oxygen defects. This also suggested a strong relationship between N doping and O defects. Notably, after the introduction of oxygen defects, the area of the peak corresponding to O_A_ at approximately 531.9 eV significantly increased. This indicates that the creation of oxygen defect sites facilitates the adsorption of oxygen-containing species such as O_2_, promoting the generation of highly oxidative ROS. The N 1s spectra of the BVO-N and BVO-N-OV samples (Figure 2b) presented two peaks at binding energies of approximately 398.4 and 399.9 eV, corresponding to lattice N atoms and chemically adsorbed N atoms, respectively [40]. This result further confirmed that N atoms were effectively integrated into the BiVO_4_ nanosheet lattice through ammonium oxalate-assisted thermal treatment and NH_3_ atmosphere heat treatment. The Bi 4f XPS spectra of all samples (Figure 2c) showed two peaks corresponding to the Bi 4f_7/2_ and Bi 4f_5/2_ signals of Bi^3+^ in BiVO_4_. The Bi 4f peaks for BVO located at approximately 159.1 and 164.3 eV. By contrast, the Bi 4f XPS peaks for the BVO-OV, BVO-N, and BVO-N-OV samples shifted slightly to higher binding energies. This shift could be due to lattice distortion resulting from N doping and oxygen defects [21,41]. Similarly, the V 2p XPS spectra of all samples (Figure 2d) showed two peaks at approximately 516.8 and 524.1 eV, which were ascribed to the V 2p_1/2_ and V 2p_3/2_ signals of BiVO_4_, respectively. Furthermore, the V 2p XPS peaks for the BVO-OV, BVO-N, and BVO-N-OV samples also shifted to higher binding energies than the BVO sample. The slight increase in the binding energies of the Bi 4f and V 2p peaks for the BVO-OV, BVO-N, and BVO-N-OV samples can be attributed to a decrease in electron cloud density, which is caused by the effects of N doping and oxygen defects [41]. The presence and generation of defects in the materials were further confirmed through room-temperature Electron Paramagnetic Resonance (EPR) analysis. As shown in Figure 2e, all the samples exhibited an EPR signal at g = 2.003, confirming oxygen vacancy defects in the samples [42]. The EPR signals of the BVO-OV, BVO-N, and BVO-N-OV samples were significantly stronger than that of the BVO sample, indicating that the ammonium oxalate-assisted thermal treatment and NH_3_ atmosphere heat treatment processes could construct oxygen vacancy defects in the BiVO_4_ nanosheets, thereby increasing the concentration of oxygen vacancy defects. Moreover, the BET specific surface area of the samples was tested using N_2_ adsorption-desorption isotherms. As shown in Figure 2f, the BET specific surface areas of the BVO, BVO-OV, BVO-N, and BVO-N-OV samples were 1.15, 4.55, 2.54, and 6.84 m^2^/g, respectively. This suggested that the introduction of N doping and oxygen vacancies could significantly enhance the specific surface area. The dual defect-engineered BVO-N-OV sample exhibited the largest specific surface area among them. More surface active sites can be exposed, thus promoting the adsorption and activation of PMS molecules on the BiVO_4_ nanosheets.

**Figure 3:**
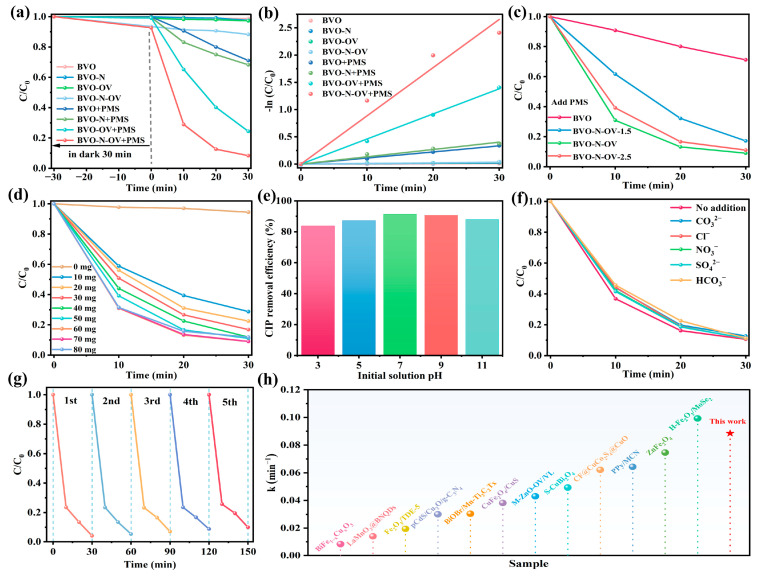
(**a**,**c**) CIP degradation experiments and (**b**) kinetic profile image in different systems. Reaction conditions: catalyst dosage: 10 mg, PMS dosage:60 mg, [CIP] = 20 ppm, [pH]_0_ = 6.5. The removal rate of CIP in different conditions, (**d**) PMS dosage; (**e**) solution pH; (**f**) the presence of inorganic anions. (**g**) Removal rate of CIP by 5 cycles of BVO-N-OV catalyst; (**h**) Comparison of the degradation rate of CIP under visible light for BVO-N-OV and other materials.

Photocatalytic degradation of CIP was used as a model reaction to explore the impact of N doping and oxygen vacancy defects on the photocatalytic activation of PMS by BiVO_4_ nanosheets. As shown in Figure 3a, all the samples demonstrated minimal photocatalytic CIP degradation performance without PMS. After adding a certain amount of PMS, the photocatalytic performance of all the samples improved significantly. This is because photocatalytic activation of PMS generates more highly oxidative ROS. It was evident that the photocatalytic performance of the dual defect-engineered BVO-N-OV sample was far superior to that of the BVO-OV, BVO-N, and BVO samples. First-order kinetic fitting was conducted for the PMS activation and CIP degradation process under visible light, as presented in Figure 3b. It was evident that the BVO-N-OV sample displayed the best photocatalytic PMS activation performance, with an apparent rate constant for CIP degradation as high as 0.08846 min^−1^ (Table S1), which was 7.9, 1.9, and 6.6 times higher than those of BVO, BVO-OV, and BVO-N, respectively. The above results indicated that N doping and oxygen vacancy defects exhibited a significant synergistic effect in promoting photocatalytic PMS activation. The coupling of doping engineering and defect engineering showed great potential for the design of efficient photocatalyst systems. In addition, the impact of the thermal treatment process on the photocatalytic PMS activation efficiency of the BVO-N-OV sample was further investigated. Figure 3c illustrated that the BVO-N-OV sample exhibited the highest photocatalytic activity when the ammonium oxalate-assisted thermal treatment time was 2 h. This might be ascribed to the optimal N doping levels and oxygen vacancy defects. Figure 3d showed that by increasing the PMS amount from 0 mg to 60 mg, under visible light irradiation within 30 min, the CIP removal rate increased from 4.0% to 91.0%. This could be explained by the fact that adding more PMS allowed for more efficient utilization of active sites, generating more SO_4_•^−^, •OH, and other ROS, thus enhancing the degradation of CIP. However, when the amount of PMS was further increased to 80 mg, the CIP degradation was slightly suppressed. This could be attributed to the catalyst’s limited active sites and the radical quenching effect from the excess PMS [43,44]. To evaluate the potential of the obtained dual defect-engineered BiVO_4_ nanosheets for treating real antibiotic wastewater, tests were conducted under various pH conditions, as shown in Figure 3e. At pH = 7.0, the BVO-N-OV sample showed the highest CIP degradation performance. When the pH was adjusted from 7.0 to 3.0 or 11.0, the CIP removal rate decreased from 91.3% to 83.8% and 87.9%, respectively. This could be due to strong acidic conditions potentially damaging the active sites on the catalyst surface, while H^+^ ions could also quench SO_4_•^−^ and •OH, thereby hindering CIP degradation. On the other hand, strong alkaline conditions significantly increased the negative charge on the catalyst surface, hindering PMS adsorption and thus limiting CIP degradation [45,46,47]. Although both acidic and alkaline conditions hindered PMS activation, the CIP removal rate still reached over 83.8% within 30 min. This indicates that the prepared BVO-N-OV photocatalyst can efficiently activate PMS over a wide pH range, making it more promising for practical applications than the traditional Fenton reaction. In real antibiotic wastewater, various inorganic ions are typically present. To further investigate the adaptability of the BVO-N-OV photocatalyst to practical application environments, the CIP removal performance was tested with different anions. As shown in Figure 3f, the addition of CO_3_^2−^, Cl^−^, NO_3_^−^, SO_4_^2−^, HCO_3_^−^, and other anions had little impact on the photocatalytic removal efficiency of CIP. This further validated the excellent adaptability of the obtained catalyst to complex real-world environments. Furthermore, the stability and reusability of the BVO-N-OV photocatalyst were assessed through cycling tests, XRD and SEM characterization. After five consecutive cycles, the CIP removal rate only showed a slight decrease and still reached 90% (Figure 3g). Meanwhile, after five cycles of reaction, the XRD pattern of the BVO-N-OV photocatalyst showed no significant changes (Figure S2a), this suggested that its crystal phase structure remained stable throughout the reaction. In addition, the SEM images (Figure S2b) showed that after five cycles of reaction, the microscopic morphology of the BVO-N-OV photocatalyst remained sheet-like, with no significant changes compared to its original state. This further verified the catalyst’s strong structural stability after repeated use. All of the above results confirmed that the constructed dual defect-engineered BVO-N-OV photocatalyst exhibited excellent stability. Satisfyingly, the photocatalytic degradation performance of BVO-N-OV for CIP was superior to most of the reported Catalyst/PMS/Vis systems (Figure 3h), further confirming the key role of the synergistic effect of N doping and oxygen vacancy defects in promoting PMS activation and highlighting the significance of our work. 

**Figure 4:**
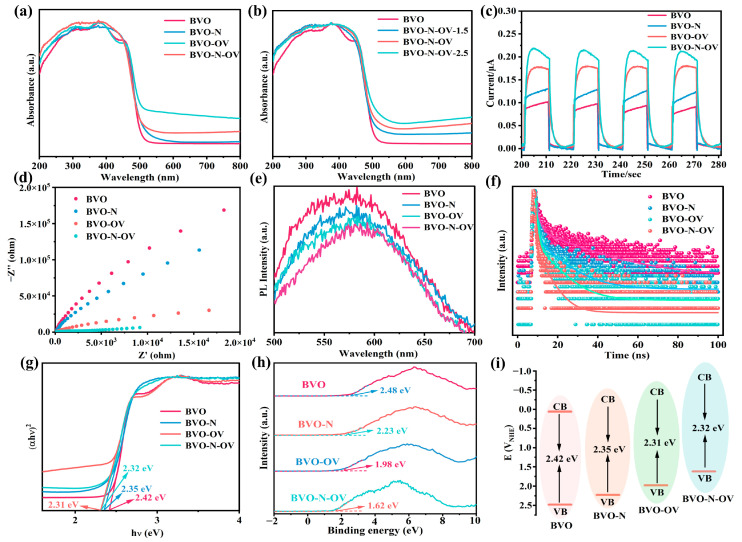
UV–vis absorption spectra of (**a**) BVO, BVO-N, BVO-OV and BVO-N-OV; (**b**) BVO and BVO-N-OV-1.5, BVO-N-OV, BVO-N-OV-2.5; (**c**) Transient photocurrent response; (**d**) EIS; (**e**) PL spectra; (**f**) TRPL spectra; (**g**) band gap energy; (**h**) VB-XPS spectra; (**i**) band structures of BVO, BVO-N, BVO-OV and BVO-N-OV.

To explain the excellent performance of the BVO-N-OV photocatalyst, a series of electrochemical characterizations were conducted. As shown in the UV-Vis diffuse reflectance spectra (DRS) of the prepared samples (Figure 4a), the absorption edge of the BVO sample was approximately 500 nm. After introducing N doping or oxygen vacancies, its visible light absorption in the 500–800 nm range was significantly enhanced. Unexpectedly, the light absorption of the BVO-N-OV sample in the 500–800 nm range was weaker than that of BVO-OV, possibly because N atoms filled some of the oxygen vacancies. Furthermore, with the extension of the ammonium oxalate-assisted thermal treatment time, the visible light absorption capacity of the resulting dual defect-engineered BiVO_4_ nanosheets gradually increased (Figure 4b), indicating that the catalyst’s light absorption performance could be regulated and tuned by adjusting the preparation conditions. The significantly enhanced visible light absorption capacity could be one of the reasons behind the BVO-N-OV photocatalyst’s superior performance. The carrier separation and transfer behaviors of the obtained catalysts were examined through transient photocurrent response, electrochemical impedance spectroscopy (EIS), photoluminescence (PL) spectra, and time-resolved photoluminescence (TRPL) spectroscopy. Figure 4c presented the transient photocurrent responses of all the samples. Relative to the BVO sample, the photocurrent density of the BVO-OV, BVO-N, and BVO-N-OV samples was significantly enhanced, and the BVO-N-OV photocatalyst exhibited the highest photocurrent density. This result indicated that the introduction of N doping or oxygen vacancy defects could effectively improve the photo-generated carrier separation, and the BVO-N-OV photocatalyst demonstrated the highest carrier separation efficiency [48]. According to the EIS curves of the samples (Figure 4d), it was observed that the Nyquist arc radius of the BVO-OV, BVO-N, and BVO-N-OV samples were much smaller than the BVO sample, and the BVO-N-OV sample exhibited the smallest Nyquist arc radius. This suggested that the interface charge transfer resistance of the BVO-OV, BVO-N, and BVO-N-OV samples was smaller than that of the BVO sample, and the introduction of dual defects was more favorable for photo-generated carrier transfer than N doping or oxygen vacancy defects alone [49]. In addition, the PL intensity of the BVO-N-OV sample was much lower than the BVO, BVO-OV, and BVO-N samples (Figure 4e), further showing that dual defects effectively restrain the recombination of photo-generated carriers [50]. Furthermore, the photo-generated carrier lifetime was examined by TRPL spectroscopy, and the results are presented in Figure 4f and Table S2. The average carrier lifetimes (τ_ave_) of the BVO, BVO-OV, BVO-N, and BVO-N-OV samples were 8.56, 5.84, 8.02, and 2.18 ns, respectively. The sharp decay of τ_ave_ indicated that the introduction of N doping and oxygen vacancy defects improved the carrier transfer efficiency of the catalyst [51,52]. All the aforementioned characterization results suggested that N doping and oxygen vacancy defects synergistically enhanced photo-generated carrier separation and migration, resulting in the excellent photocatalytic performance of the BVO-N-OV catalyst.

The band structure determines the electronic and optical properties of a material, and is a key factor influencing its photocatalytic activity and selectivity. Thus, the band gaps (Eg) of all the samples were calculated from the Tauc plots based on the Kubelka-Munk function (Figure 4g) [53]. The calculated E_g_ values of the BVO, BVO-OV, BVO-N, and BVO-N-OV samples were 2.42, 2.31, 2.35, and 2.32 eV, respectively. This result showed that the introduction of N doping and oxygen vacancy defects narrowed the band gap, thereby extending the material’s light absorption range. The energy level structure of the obtained photocatalysts was further determined using the VB-XPS spectrum. As shown in Figure 4h, the valence band (VB) potentials of the BVO, BVO-OV, BVO-N, and BVO-N-OV samples were 2.48, 1.98, 2.23, and 1.62 eV, respectively. Accordingly, the conduction band (CB) potentials of the BVO, BVO-OV, BVO-N, and BVO-N-OV samples were calculated using the equation E_CB_ = E_VB_ − E_g_, yielding values of −0.06, −0.33, −0.12, and −0.70 eV, respectively. Therefore, the energy band structures of all the samples were established and shown in Figure 4i. It was clear that after the introduction of N doping or oxygen vacancy defects, the band gap narrowed, and the conduction band potential also became more negative. Among them, the BVO-N-OV sample exhibited the most negative CB potential, which might be responsible for its excellent photocatalytic performance. These results indicated that doping and defect engineering could synergistically optimize the materials’ energy band structure, thereby enhancing the photocatalytic activity.

**Figure 5:**
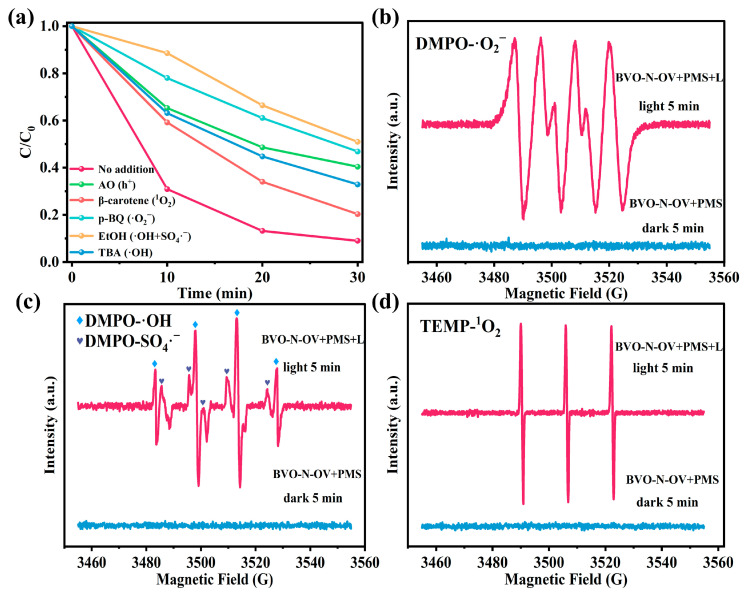
(**a**) Effect of scavengers on CIP degradation in the BVO-N-OV/PMS/Vis system; EPR spectra of (**b**) DMPO-•O_2_^−^, (**c**) DMPO-•OH/SO_4_•^−^, and (**d**) TEMP-^1^O_2_ adducts obtained for BVO-N-OV and PMS under darkness and irradiation.

**Scheme 2:**
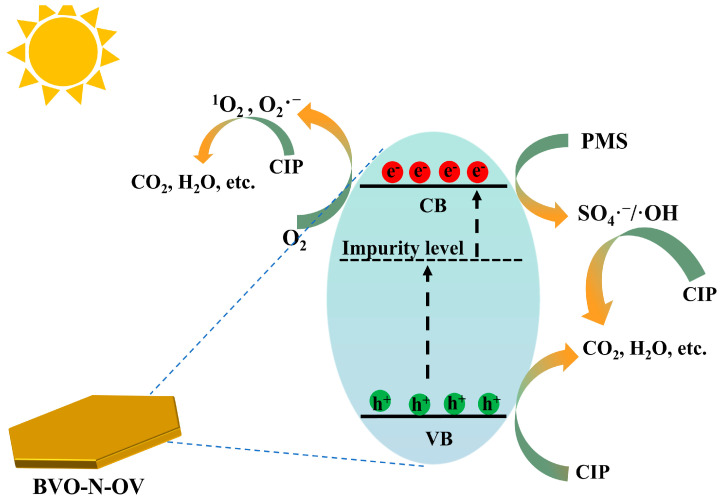
The proposed mechanism for the photocatalytic activation of PMS through BVO-N-OV process.

To investigate the carrier migration behavior of the BVO-N-OV photocatalyst and the PMS activation pathway and reveal the CIP degradation mechanism, active species trapping tests were carried out to identify the primary reactive species in the CIP degradation process. As shown in Figure 5a, when ethanol (EtOH) and tert-butyl alcohol (TBA) were added as SO_4_•^−^ and •OH scavengers into the BVO-N-OV/PMS/Vis system [8,44], CIP removal rate dropped from 91% to 50% and 70%, respectively. This suggested that SO_4_•^−^ and •OH were crucial in the CIP degradation. When β-carotene and p-benzoquinone (p-BQ) were introduced as scavengers for ^1^O_2_ and •O_2_^−^, the CIP removal rate dropped from 91% to 80% and 56%, respectively. This highlighted the crucial role of ^1^O_2_ and •O_2_^−^ in the CIP removal process, with •O_2_^−^ having a more significant impact than ^1^O_2_. When oxalic acid (AO) was added to the system as a hole (h^+^) scavenger, the removal rate of CIP sharply decreased from 91% to 60%, indicating that h^+^ might be crucial to the CIP degradation process. To further validate the results of the active species trapping experiments, EPR was used to study free radical generation during the reaction in the BVO-N-OV/PMS system. As seen in Figure 5b–d, although the BVO-N-OV/PMS system was able to generate DMPO-•O_2_^−^, DMPO-•OH, DMPO-SO_4_•^−^ and TEMP-^1^O_2_ signals under dark conditions [8,44,54,55,56], the signal intensities were very weak. This indicated that the BVO-N-OV/PMS system generated only a limited number of free radicals in the dark. As expected, after 5 min of visible light irradiation, the signal intensities of all free radicals in the BVO-N-OV/PMS system were significantly enhanced, indicating the key role of the photocatalytic effect in the free radical generation process. The significantly enhanced free radical generation level could explain the excellent CIP removal performance of the BVO-N-OV/PMS/Vis system. Based on the experimental findings and analysis above, a potential reaction mechanism is suggested (Scheme 2). Visible light irradiation led to the generation of photoelectrons and holes in BVO-N-OV. The photo-induced electrons were excited to the CB, while the photo-induced holes remained in the valence band VB. The introduction of N doping and oxygen vacancy defects created an impurity energy level within the band gap, enhancing light absorption and facilitating the temporary storage of the excited electrons, suppressing their recombination with the photo-induced holes. The electrons in the CB of BiVO_4_ nanosheets reacted with PMS and dissolved O_2_ to generate SO_4_•^−^, •OH, •O_2_^−^, and ^1^O_2_. The more negative CB potential of BVO-N-OV aided PMS activation, leading to the generation of more ROS. The generated ROS oxidized and decomposed CIP into small molecular products such as H_2_O and CO_2_. The VB potential of BiVO_4_ nanosheets (+1.62 eV) is too negative to oxidize OH^−^ into •OH. Even so, the photo-induced h^+^ has a strong oxidizing ability and could directly oxidize CIP into small molecular products such as H_2_O and CO_2_. In a word, the construction of dual defects synergistically promoted the photocatalytic activation of PMS and the multipath generation of ROS, achieving efficient CIP removal.

## 4. Conclusions

In conclusion, we successfully constructed dual defect-engineered BiVO_4_ nanosheets by introducing N-doping and oxygen vacancies through ammonium oxalate-assisted thermal treatment. The prepared BVO-N-OV photocatalyst efficiently activated PMS under visible light irradiation, achieving a 91.0% removal rate of CIP within 30 min. The apparent rate constants for CIP removal in the BVO-N-OV/PMS/Vis system were 7.9, 1.9, and 6.6 times those of BVO, BVO-OV, and BVO-N, respectively. More importantly, even under a wide pH range (3.0–11.0) and with different anions, the BVO-N-OV/PMS/Vis system still demonstrated stable and excellent CIP removal performance. The results of active species trapping experiments and EPR tests confirmed that the key reactive species involved in the CIP degradation process include SO_4_•^−^, •OH, •O_2_^−^, ^1^O_2_ and h^+^. The excellent photocatalytic performance of the BVO-N-OV/PMS/Vis system could be attributed to the following factors: (1) BiVO_4_ has a relatively wide visible light absorption range. Its band structure enables it to effectively absorb visible light and generate photoexcited carriers (electrons and holes). (2) N doping and oxygen vacancy defects synergistically optimized the energy band structure, enhancing visible light absorption and promoting the separation of photo-generated carriers; (3) The introduction of oxygen vacancies and N doping optimized the surface properties, supplying numerous active sites that accelerated the adsorption and activation of PMS molecules. This research is anticipated to offer valuable insights into the coupling mechanism of defect engineering and doping engineering in photocatalytic PMS activation and new ideas for developing and fabricating efficient photocatalysts/PMS systems for wastewater treatment.

## Data Availability

No additional data are available.

## Supplementary Materials

The following supporting information can be downloaded at: https://www.mdpi.com/article/10.3390/nano15050373/s1, Figure S1: EDX elemental mapping profiles of BVO-N; Figure S2: (a) XRD patterns and of BVO-N-OV before and after photocatalytic reaction; (b) SEM image of BVO-N-OV after photocatalytic reaction; Table S1: The rate constants k for activating PMS to degrade CIP by BVO, BVO-N, BVO-OV and BVO-N-OV; Table S2: The transient fluorescence lifetimes of BVO, BVO-N, BVO-OV and BVO-N-OV.
