# Supplementary material for: Dual Defect-Engineered BiVO4 Nanosheets for Efficient Peroxymonosulfate Activation
Source: Nanomaterials (Basel). 2025 Feb 28;15(5):373. doi: 10.3390/nano15050373 (PMC11902240; doi:10.3390/nano15050373)
Supplement: Supplementary file 1 [file nanomaterials-15-00373-s001.zip › nanomaterials-3491874-supplementary.pdf]

Supporting Information

Dual defect-engineered $BiVO_4$ nanosheets for efficient peroxymonosulfate activation

Jiabao Wu[1], Meiyu Xu[2], Zhenzi Li[2], Mingxia Li[1,*], Wei Zhou[2,*]

[1] School of Chemistry and Materials Science, Key Laboratory of Functional Inorganic Material Chemistry, Ministry of Education of the People's Republic of China, Heilongjiang University, Harbin 150080, P. R. China
[2] Shandong Provincial Key Laboratory of Molecular Engineering, School of Chemistry and Chemical Engineering, Qilu University of Technology (Shandong Academy of Sciences), Jinan, Shandong, 250353, P. R. China
* Correspondence: limingxia@hlju.edu.cn; zwchem@hotmail.com

1. Experimental section

1.1 Characterizations

The X-ray diffraction (XRD) patterns of BVO, BVO-OV, BVO-N and BVO-N-OV were collected using a MiniFlex X-ray diffractometer with a Cu Kα radiation source (1.54 Å). Filed emission scanning electron microscopy (FE-SEM, JEOL-6500) and transmission electron microscopy (TEM, JEOL-2100F) were used to study the morphology. X-ray photoelectron spectroscopy (XPS, Thermo Scientific ESCALAB 250Xi) was used to investigate the elemental composition and surface valence states of the samples. Oxygen vacancies in the samples were determined by electron spin resonance (ESR, JES-FA300). The Brunauer-Emmett-Teller (BET) specific surface area of the samples was determined using a surface area analyzer (Micromeritics ASAP 2460) with nitrogen adsorption-desorption method. Using $BaSO_4$ as a background reference, the UV diffuse reflectance spectra of the samples were obtained on the UV-2550 (PerkinElmer) spectrometer, which reflected the optical properties of the samples. Time-resolved photocurrent behavior and electrochemical impedance spectroscopy (EIS) were conducted using a three-electrode configuration on an electrochemical workstation (VersaSTAT 4). An FTO glass with an area of 1×1 cm^2 served as the working electrode, with an Ag/AgCl electrode as the reference electrode and a platinum slice as the counter electrode in a 0.1 M Na_2SO_4 aqueous solution. The

photoluminescence (PL) emission spectra were measured using a fluorescence spectrometer (Edinburgh FLS1000) with an excitation wavelength of 437 nm. Under illumination with a 300 W xenon lamp and a Cut 420 filter, electron spin resonance (ESR) spectra were recorded on a Bruker A300-10/12 spectrometer using 5,5-dimethyl-1-pyrroline N-oxide (DMPO) and 2,2,6,6-tetramethylpiperidine (TEMP) as spin trapping agents.

1.2 Photocatalytic activity measurements

In a typical PMS catalytic activation experiment, 10 mg of the catalyst was dispersed in 60 mL of 20 ppm ciprofloxacin (CIP) solution and sonicated for 30 s. Then, the mixture was stirred in the dark using a magnetic stirrer for 30 min to ensure adsorption-desorption equilibrium was reached. Afterward, a certain amount of PMS was added to the solution, and the mixture was exposed to light. Samples were taken at fixed time intervals, transferred into two 2.5 mL centrifuge tubes, and centrifuged to remove the catalyst. After collecting the supernatant, it was transferred into a quartz cuvette, and the concentration of pollutants was analyzed using a UV-Vis spectrophotometer (UV-9000S, Metash).

2. Supporting Figures and Table

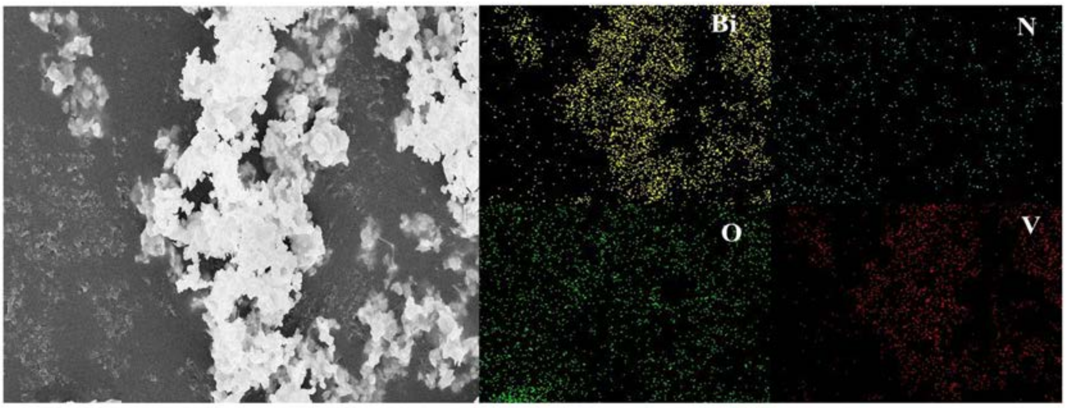

Figure S1. EDX elemental mapping profiles of BVO-N.

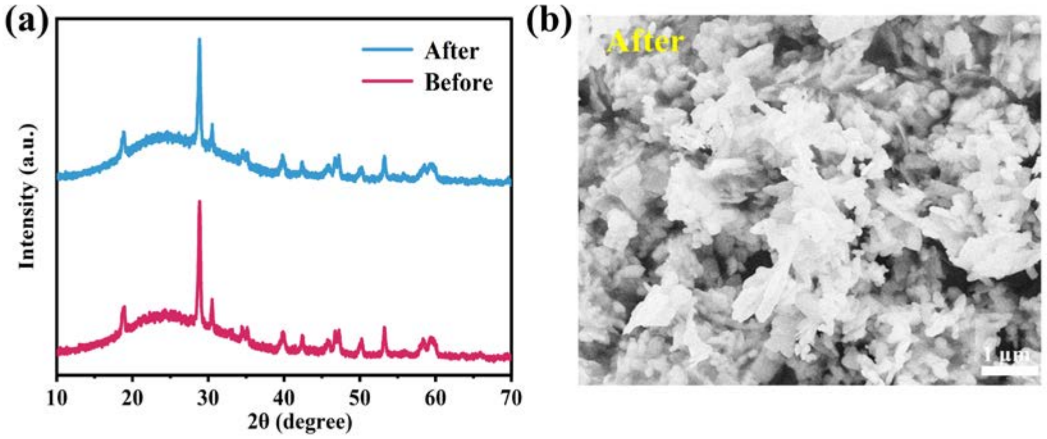

Figure S2. a) XRD patterns and of BVO-N-OV before and after photocatalytic reaction; b) SEM image of BVO-N-OV after photocatalytic reaction.

Table S1. The rate constants k for activating PMS to degrade CIP by BVO, BVO-N, BVO-OV and BVO-N-OV.

**Sample**	BVO	BVO-N	BVO-OV	BVO-N-OV
k (min^{-1})	0.01115	0.01341	0.04602	0.08846

Table S2. The transient fluorescence lifetimes of BVO, BVO-N, BVO-OV and BVO-N-OV.

**Sample**	τ_1	τ_2	$\tau_{average}$
BVO	1.40 ns	23.3 ns	8.56 ns
BVO-N	1.04 ns	12.94 ns	8.02 ns
BVO-OV	1.39 ns	10.48 ns	5.84 ns
BVO-N-OV	0.85 ns	6.49 ns	2.18 ns
